# Left Atrial Dissection After Mitral and Aortic Valve Replacement: The Importance of Early Diagnosis of a Rare Entity

**DOI:** 10.3390/reports8040205

**Published:** 2025-10-17

**Authors:** Despoina Sarridou, Sophia Anastasia Mouratoglou, Rafail Ioannidis, Aikaterini Amaniti, Giakoumis Mitos, Eleni Argiriadou

**Affiliations:** 1 Department of Anesthesiology and Intensive Care, AHEPA University Hospital of Thessaloniki, 54124 Thessaloniki, Greece; dodasarri@yahoo.gr (D.S.); amanitik@gmail.com (A.A.); jacomomitos@yahoo.gr (G.M.); argiriadouhelena@hotmail.com (E.A.); 2 3rd Department of Cardiology of Aristotle University of Thessaloniki, Hippocrates General Hospital of Thessaloniki, 54642 Thessaloniki, Greece; s_mouratoglou@yahoo.gr; 3 Department of Anesthesiology and Pain Medicine, General Hospital of Drama, 66100 Drama, Greece

**Keywords:** atrial dissection, calcification, mitral valve

## Abstract

**Background and Clinical Significance:** Left atrial dissection is a rare surgical complication (occurring in 0.16% of cases), which results in the formation of a large cavity between the endocardium and the epicardium. **Case Presentation**: We report a case of a 78-year-old man who underwent combined aortic and mitral valve replacement. Extensive debridement of the calcified mitral annulus most probably precipitated the formation of a dissection flap detected by transesophageal echocardiography after protamine administration. Profound hypotension and hemodynamic instability were treated with inotropic and vasopressor support and fluid administration without any further surgical intervention. **Conclusions**: The patient recovered uneventfully under conservative management, highlighting the importance of perioperative echocardiography for prompt diagnosis and tailored intervention.

## 1. Introduction and Clinical Significance

Left atrial dissection after cardiac surgery is a rare complication that may affect the outcome after cardiac surgery. Left atrial dissection after mitral valve replacement is an extremely rare but recognized complication with a reported incidence rate reaching 0.16%. The dissection creates a large cavity between the endocardium and epicardium, practically giving the impression of two chambers in the left atrium of the heart [1]. Technical difficulties with the removal of the native valve may lead to difficult extraction, and preexisting annular calcification is another recognized factor associated with the aforementioned complication.

## 2. Case Presentation

We present the case of a male 78-year-old patient with known aortic stenosis. The patient presented for an elective aortic valve replacement. On his regular preoperative workup, a new transthoracic echocardiography (TTE) study revealed mitral valve pathology requiring intervention too. The echocardiography study showed good left and right ventricular function, severe aortic stenosis with mild regurgitation, moderate mitral valve stenosis, and mitral regurgitation. There were also signs of pulmonary hypertension. From his previous medical history, the patient also suffered from arterial hypertension. A written consent was obtained for both the procedure and the presentation of the case.

**Figure 1:**
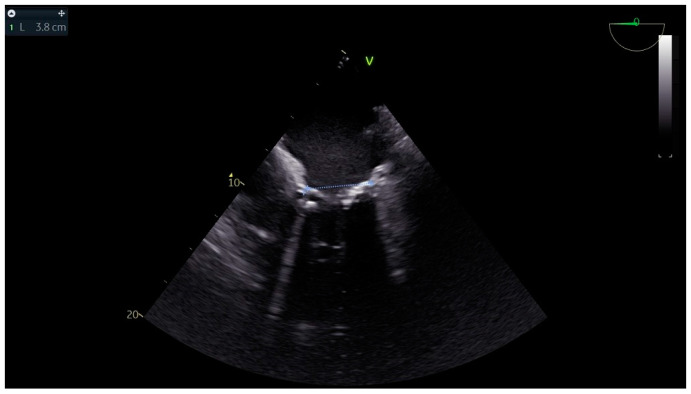
TEE preoperative image showing mitral valve calcification with size of 38 mm.

**Figure 2:**
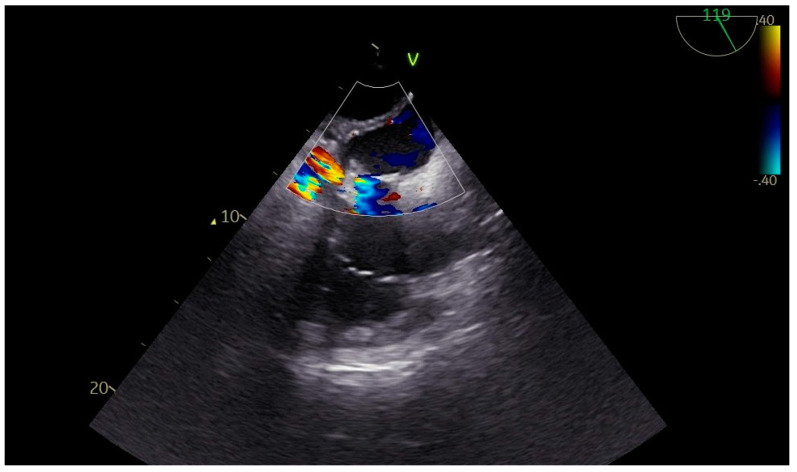
Left atrial dissection flap.

**Figure 3:**
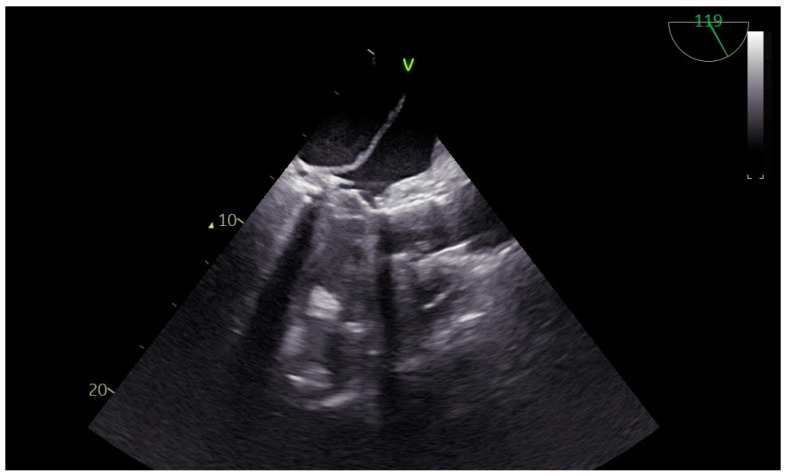
Color flow Doppler showing a competent prosthetic mitral valve in place with dissection flap in the left atrium.

**Figure 4:**
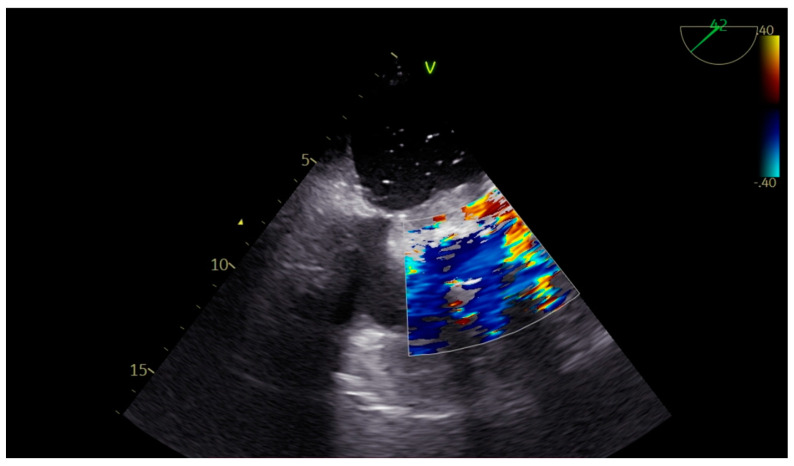
Postoperative Perceval well-sited mitral valve replacement.

Advanced hemodynamic monitoring as per local protocol was applied with cannulation of the right radial and femoral arteries as well as establishment of peripheral access with two (wide) 16 G cannulae. Baseline invasive blood pressure before induction was 145/60 mmHg. Induction to anesthesia was uneventful without any signs of hemodynamic instability, using balanced anesthetics for the maintenance of general anesthesia too. A central line and pulmonary artery catheter were placed in the right internal jugular vein. Continuous cardiac monitoring and cerebral oximetry monitoring were applied while depth of anesthesia was monitored with the Bispectral Index. Intraoperative transesophageal echocardiography (TEE) was used and confirmed the findings of TTE, especially with a heavily calcified mitral valve annulus (Figure 1). Standard bicaval extracorporeal circulation through sternotomy and mild systemic hypothermia (32 °C) were used. Myocardial protection was made by a combination of antegrade and retrograde cold hyperkalemic blood cardioplegia. Cardiopulmonary bypass time was nearly two hours. The separation from cardiopulmonary bypass was initially smooth with minimal vasopressor support (blood pressure > 85–90/45 mmHg). A few minutes after the protamine administration an episode of profound hypotension caused major hemodynamic instability (blood pressure > 55–60/35 mmHg), which was related to the protamine side effects. However, since it persisted, regardless of the treatment with vasopressors, a thorough reassessment with the TEE was performed. This dynamic investigation revealed an obvious flap fluctuating in the left atrium, suggestive of a dissection flap in the chamber (Figure 2). (The dissection flap size was at least 7.2 cm. There was difficulty in the precise measurement of its dimension because the left atrium roof could not be visualized.) Otherwise, the two new prosthetic valves were well seated without any residual regurgitation (Figure 3 and Figure 4). The most plausible explanation for the presence of the dissection flap was the earnest mitral valve replacement performed in the setting of extensive mitral annular calcification. Hemodynamic stability was achieved with boluses of vasopressors (phenylephrine) and noradrenaline and adrenaline infusions. Since the patient was relatively stabilized under this support, the chest was closed and he was taken to the intensive care unit with a target for systolic blood pressure less than 100–120 mmHg for the first twenty-four hours (Table 1).

A sutureless rapid-deployment Perceval M (LivaNova, London, United Kingdom) aortic valve bioprosthesis was implanted through an aortic transverse incision after performing annular debridement of all calcification. The mitral valve was exposed by opening the dome of the left atrium dissecting the Sondergaard’s groove. Severe calcification of the mitral annulus with extension to the myocardium and the papillary muscles was identified. Debridement of all calcification was attempted, leaving the calcified parts penetrating the cardiac muscle in order to avoid the risk of rupturing the left ventricular wall. A Mosaic™ mitral valve bioprosthesis (31mm, Medtronic, Inc.; Minneapolis, Minn) was implanted with interrupted sutures of 2-0 Ethibond Excel^®^ (Ethicon, Johnson & Johnson company; Somerville, NJ, USA) and reinforced by subannular Teflon felt pledgets. Destruction of the annular anatomy during debridement in the area between 8 and 11 o’clock made suture positioning extremely challenging. This was most likely the site from where the dissection may have originated.

After weaning from mechanical ventilation, the patient was extubated the day after his procedure and he remained stable on minimum vasopressor support. His ICU and hospital stay was uneventful. His TTE findings remained unchanged, showing preserved left ventricular function and well-seated valves with the same left atrium images and the flap present. He was discharged on Day 10 postoperatively without any significant co-morbidities. His 30-day follow-up was also unremarkable.

## 3. Discussion

Left atrial dissection is a quite rare entity and different mechanisms seem to be related to its etiology. Debridement of heavy calcification, excessive traction of sutures in the annulus disrupting and tearing the tissues, and improper handling of the annulus with a prosthesis mismatch may be responsible for the complication. In our case, left atrial dissection was associated with an extremely (copious) difficult debridement of heavy calcification of the annulus. Nevertheless, it was treated conservatively and the patient had an uneventful recovery period in the cardiothoracic intensive care unit. Hemodynamic instability, especially during the immediate period after the separation from cardiopulmonary bypass and also during the whole postoperative phase, mandates re-exploration and surgical intervention [2]. The role of perioperative TEE is of significant importance in the early recognition of the aforementioned complication and has been highlighted from other authors too. Left atrial dissection may also occur after excision of cardiac masses originating by the atrial walls such as myxomas [3]. It can even more rarely be associated with coronary bypass grafting or aortic valve surgery [4].

Repeat imaging for follow-up on the following days was deemed necessary in order to reassess for any changes with regard to the original findings. Echocardiography findings remained stable and confirmed the presence of a dissection flap creating two chambers in the left atrium. Another interesting case in the literature suggests improvement in the dissection with vanishing of the endocardial tissue which splits the cavity in two chambers, leading to spontaneous resolution of the complication on the second postoperative day. The same study also highlights the importance of the transesophageal imaging to the early diagnosis [5]. Prompt diagnosis with intraoperative imaging can potentially dictate the pathology and guide a successful immediate intervention, avoiding a second cardiac surgery with increased mortality. The majority of cases in the literature are characterized by a lack of hemodynamic compromise and were treated conservatively, especially when separation from cardiopulmonary bypass was uneventful. Healing and remodeling of the atrium is possible and well recognized [4]. Other contributing factors could be related to a background of infected endocarditis or connective tissue disease like systemic sclerosis, as suggested from other case reports [6,7]. Even though our patient had healthy tissues, he suffered from heavy calcification of the mitral valve apparatus and annulus. In general, tight hemodynamic control and deep sedation is advised especially for the first postoperative hours in order to avoid surges in the blood pressure and potential rupture. Further imaging is strongly suggested as well. Differential diagnosis for acute hemodynamic instability on the immediate post-CPB period includes conditions such as acute right ventricular failure, vasoplegia, hypotension due to protamine reaction and surgical bleeding, or coagulation disorders [8,9,10,11]. Alternative management could have been the reopening of the heart chambers for re-exploration, but this would complicate the case with a second round of CPB, leading to a bad outcome. Our patient had preserved right and left ventricular function and surgical bleeding was under control.

## 4. Conclusions

The use of a recent preoperative workup with a new TTE study revealed the mitral valve pathology which required intervention. In addition to this, the use of intraoperative TEE allowed the early recognition of the complication involving the left atrium dissection. The use of TEE is undoubtedly of great value and very useful especially in the immediate post-bypass period—a time frame conducive to hemodynamic instability. Also, in the presence of hemodynamic instability, the possibility of atrial dissection should be considered and confirmed with TEE.

## Figures and Tables

**Table 1:** The preoperative echocardiography values and dimensions.

Left Atrium Dilated	>6.5 × 5.2 mm (No difficulty in visualization of the left atrial roof). No spontaneous echocardiography contrast seen. Left atrial appendage: No signs of clots.
Mitral valve annulus	38 mm
Aortic Valve Annulus	23 mm with heavy calcification
Sinotubular Junction	26 mm
Ascending Aorta	32 mm
LVEF	55%
LVIDd	55 mm
IVSd	13 mm
LVPWd	15 mm
TAPSE	19 mm

## Data Availability

The original contributions presented in this study are included in the article. Further inquiries can be directed to the corresponding authors.

## Abbreviations

The following abbreviations are used in this manuscript:

TTE	Transthoracic echocardiography
TEE	Transesophageal echocardiography
LVIDd	Left ventricular internal end diastolic diameter
IVSd	Linear dichroism
LVPWd	Left Ventricular Posterior Wall thickness in diastole
TAPSE	Tricuspid Annular Plane Systolic Excursion
ICU	Intensive care unit
CPB	Cardiopulmonary bypass
